# Galectin-1 deficiency attenuates imiquimod-induced psoriasiform skin inflammation and is associated with reduced macrophage recruitment

**DOI:** 10.3389/fimmu.2026.1960116

**Published:** 2026-09-30

**Authors:** Shiu-Ju Yang, Shih-Yao Chen, Chia-Hsin Hou, Chao-Liang Wu, Ming-Fei Liu, Yuh-Ling Chen, Tak-Wah Wong, Chuang-Ming Wang, Ai-Li Shiau

**Affiliations:** 1 Department of Microbiology and Immunology, National Cheng Kung University Medical College, Tainan, Taiwan; 2 Institute of Infectious Diseases and Vaccinology, National Health Research Institutes, Miaoli, Taiwan; 3 Department of Nursing, College of Nursing, Chung Hwa University of Medical Technology, Tainan, Taiwan; 4 Department of Orthopedics, College of Medicine, National Cheng Kung University, Tainan, Taiwan; 5 Department of Pediatrics, Ditmanson Medical Foundation Chia-Yi Christian Hospital, Chiayi, Taiwan; 6 Department of Medical Research, Ditmanson Medical Foundation Chia-Yi Christian Hospital, Chiayi, Taiwan; 7 Department of Biochemistry and Molecular Biology, National Cheng Kung University Medical College, Tainan, Taiwan; 8 Department of Internal Medicine, National Cheng Kung University Medical College, Tainan, Taiwan; 9 Institute of Oral Medicine, National Cheng Kung University Medical College, Tainan, Taiwan; 10 Department of Dermatology, National Cheng Kung University Medical College and Hospital, Tainan, Taiwan

**Keywords:** CXCL10, galectin-1, imiquimod, macrophage migration, psoriasis, skin inflammation

## Abstract

**Introduction:**

Psoriasis is an immune-mediated inflammatory skin disease in which innate immune activation and the IL-23/IL-17 axis coordinate keratinocyte hyperproliferation and leukocyte recruitment. Galectin-1 (Gal-1) is a glycanbinding protein with context-dependent immunoregulatory effects; however, its function in psoriasiform skin inflammation remains incompletely defined. Methods: Here, we evaluated Gal-1 expression in human psoriasis lesions and investigated its contribution to imiquimod (IMQ)-induced psoriasiform inflammation in mice.

**Results:**

Gal-1 immunoreactivity was increased in lesional skin from patients with psoriasis and in IMQ-treated wildtype mice. Compared with IMQ-treated wild-type mice, Gal-1-deficient mice developed less severe erythema, scaling, epidermal thickening, acanthosis, and parakeratosis. Attenuated disease was accompanied by reduced lymphoidorgan enlargement and lower frequencies or numbers of selected innate and adaptive immune-cell populations. In skin, Gal-1 deficiency was associated with reduced macrophage accumulation and diminished CXCL10 mRNA expression, whereas several other inflammatory transcripts showed variable, non-significant trends. In vitro, macrophages from Gal-1 deficient mice displayed impaired migration, while recombinant Gal-1 enhanced macrophage migration in cells from both genotypes.

**Conclusion:**

These findings support Gal-1 as a potential therapeutic target for modulating inflammatory-cell recruitment and alleviating psoriasiform skin inflammation.

## Graphical abstract

**Figure:**
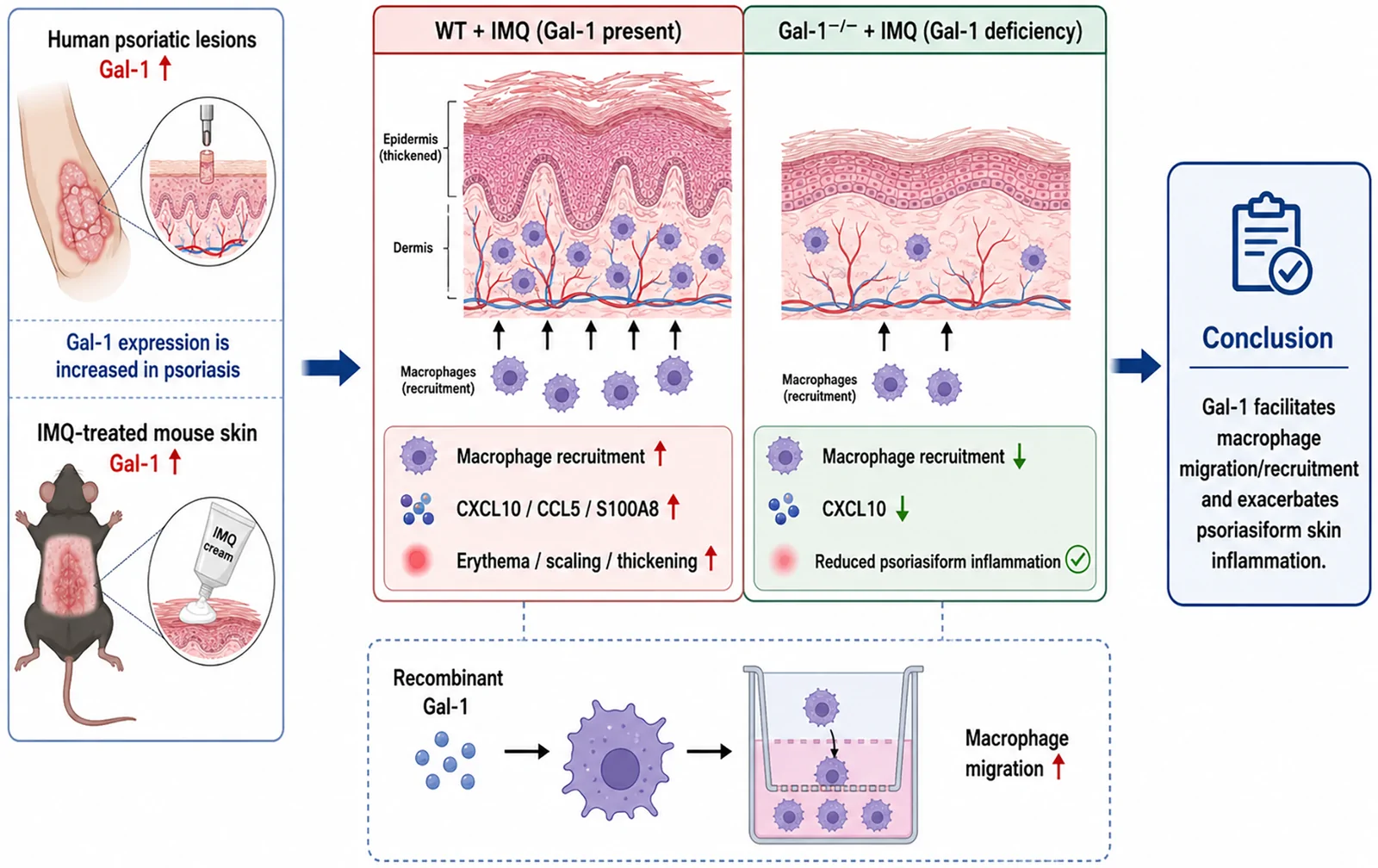
Galectin-1 deficiency attenuates imiquimod-induced psoriasiform skin inflammation and is associated with reduced macrophage recruitment. Galectin-1 (Gal-1) expression was increased in human psoriatic lesions and in dorsal skin from imiquimod (IMQ)-treated mice. In wild-type mice, IMQ treatment induced marked epidermal thickening, erythema, scaling, inflammatory-cell infiltration, and increased expression of CXCL10, CCL5, and S100A8. In contrast, Gal-1 deficiency attenuated the clinical and histological severity of psoriasiform skin inflammation and was associated with reduced macrophage accumulation and lower CXCL10 expression. *In vitro*, recombinant Gal-1 enhanced macrophage migration in transwell assays. Collectively, these findings demonstrate that Gal-1 deficiency is associated with attenuated psoriasiform skin inflammation, reduced macrophage accumulation, and altered macrophage migratory responses.

## Highlights

- Galectin-1 expression was increased in human psoriatic lesions and in imiquimod-treated mouse skin.
- Galectin-1 deficiency attenuated clinical and histological features of imiquimod-induced psoriasiform inflammation.
- Reduced skin macrophage accumulation and lower CXCL10 expression accompanied Galectin-1 deficiency.
- Recombinant Galectin-1 enhanced macrophage migration *in vitro*.

## 1 Introduction

Psoriasis is a chronic immune-mediated inflammatory disease characterized by erythematous, scaly plaques, epidermal hyperplasia, and infiltration of innate and adaptive immune cells (1, 2). Activated T cells are prominent in psoriatic lesions, and the IL-23/IL-17 axis, together with IFN-γ- and TNF-driven inflammatory programs, plays a central role in disease maintenance and tissue remodeling (3–5). Although biologics that target these pathways have transformed clinical management, the tissue-specific mechanisms that control immune-cell recruitment and persistence remain incompletely understood (6, 7).

Galectin-1 (Gal-1) is a β-galactoside-binding lectin that regulates cell survival, adhesion, migration, and immune responses. Depending on the cellular and inflammatory context, Gal-1 can induce apoptosis in activated T cells, promote tolerogenic circuits, or facilitate the migration of myeloid and stromal cells (8–10). Gal-1 has also been implicated in cell adhesion, angiogenesis, mRNA processing, and differentiation programs, underscoring its broad and context-dependent biological functions (11–13). Experimental studies further indicate that Gal-1 can modulate the migration of monocytes, dendritic cells, neutrophils, retinal epithelial cells, and myofibroblasts (14–18). Our previous studies demonstrated that Gal-1 induces T-cell apoptosis in rheumatoid arthritis (19, 20). Collectively, these pleiotropic activities make it difficult to predict whether Gal-1 is protective or pathogenic in a given inflammatory disease.

In this study, we aimed to examine Gal-1 expression in human psoriatic lesions and use the imiquimod (IMQ)-induced mouse model (21), which captures important IL-23/IL-17-driven features of psoriasiform skin inflammation, to test whether Gal-1 contributes to disease development (22). We further assessed systemic and cutaneous immune changes, inflammatory gene expression, and macrophage functions. Our data indicate that Gal-1 deficiency attenuates IMQ-induced disease and is associated with reduced macrophage accumulation and reduced CXCL10 expression.

## 2 Materials and methods

### 2.1 Human skin specimens

Human skin samples were collected from outpatients at the Department of Dermatology, National Cheng Kung University Hospital. Patients with psoriasis vulgaris (n = 21) and healthy control participants undergoing excision of benign skin tumors (n = 7) were enrolled according to the criteria approved by the Human Research Ethics Committee of National Cheng Kung University Hospital (**Table 1**). Control skin samples included four skin tags and one nevus lipomatosus. These specimens were selected because the adjacent skin appeared normal or near normal and showed no histopathological evidence of inflammation. All study procedures were approved by the Institutional Review Board of National Cheng Kung University Hospital. There were no significant differences in age distribution or disease duration between the groups (p > 0.05, Student’s *t*-test). All tissue processing was performed at the Department of Pathology and Tissue Bank, National Cheng Kung University Hospital.

**Table 1:** Demographic data of patients.

Group	Age (years)	Duration of skin lesions (years)
Benign tumors without inflammation (n = 7)	32 – 60, mean 49 ± 11	2 – 20, mean 11 ± 8
Psoriasis (n = 21)	18 – 69, mean 44 ± 18	0.4 – 15, mean 6 ± 6

Human skin samples were obtained from healthy control participants undergoing excision of benign skin tumors (n = 7) and from lesional skin of patients with psoriasis vulgaris (n = 21; **Table 1**). The control specimens were selected because the surrounding skin appeared normal or near normal and showed no histopathological evidence of inflammation. All study procedures were approved by the Institutional Review Board. There were no significant differences in age distribution or disease duration between the two groups. Data are presented as the mean ± SEM, and comparisons were performed using Student’s t-test (p > 0.05).

### 2.2 Mice and induction of psoriasiform dermatitis

C57BL/6 (B6) mice were obtained from the National Laboratory Animal Center (Taipei, Taiwan). Gal-1-deficient mice (14) and wild-type littermate or B6 controls were maintained under specific-pathogen-free conditions in individually ventilated cages at the Laboratory Animal Center, National Cheng Kung University. At 8–10 weeks of age, dorsal hair was shaved and mice received 62.5 mg of 5% IMQ cream (Aldara; 3M Pharmaceuticals) on the back skin once daily for six consecutive days, as described previously (21). Untreated genotype-matched mice served as controls. All of the experiments were approved by the animal ethics committee and performed according to institutional guidelines.

### 2.3 Clinical scoring and histological assessment

Dorsal-skin erythema, scaling, and thickening were scored daily using a modified Psoriasis Area and Severity Index (PASI), with each component graded from 0 to 4 (0, none; 1, slight; 2, moderate; 3, marked; 4, very marked) (21). Skin specimens collected on day 6 were formalin-fixed, paraffin-embedded, and stained with hematoxylin and eosin (H&E). Epidermal thickness and/or keratinocyte-layer number were quantified from predefined microscopic fields by an investigator blinded to group allocation.

### 2.4 Immunohistochemistry

Paraffin sections were deparaffinized, rehydrated, and subjected to heat-induced antigen retrieval in 10 mM citrate buffer (pH 6.0). Sections were incubated with antibodies against CD49b (clone DX5; BioLegend, San Diego, CA, USA), Mac-3 (clone M3/84; BD Pharmingen, San Diego, CA, USA), and pDC/IPC (clone 120G8.04; Dendritics, San Diego, CA, USA), followed by the LSAB + System-HRP (Dako, Carpinteria, CA, USA) according to the manufacturer’s instructions and counterstained with hematoxylin. Positive cells were quantified in predefined fields and normalized to tissue area or epidermal length, as appropriate.

### 2.5 Flow cytometry

Single-cell suspensions were prepared from draining lymph nodes and spleens by mechanical disruption and filtration through a 60-μm mesh. After Fc-receptor blockade, cells were stained with fluorochrome-conjugated antibodies against CD4 (clone GK1.5; BD Pharmingen), CD49b (clone DX5; BD Pharmingen), F4/80 (clone BM8; eBioscience, San Diego, CA, USA), and pDC markers(clone 120G8.04; Dendritics). For intracellular cytokine and transcription-factor staining, cells were fixed, permeabilized, and stained for IFN-γ (clone XMG1.2; eBioscience), IL-4 (clone 11B11; eBioscience), IL-17A (clone eBio17B7; eBioscience), and Foxp3(clone MF23; BD Pharmingen). Samples were acquired using a FACSCalibur system and analyzed with CellQuest software (BD Bioscience).

### 2.6 NK-cell cytotoxicity, phagocytosis, and transwell migration

Cytotoxicity was assessed using a lactate dehydrogenase (LDH) release assay. Mice were sacrificed, and single-cell suspensions of splenocytes were prepared. Splenocytes at various concentrations were used as effector cells and co-cultured with YAC-1 target cells (1 × 10^4^ cells/well) in 100 μL of RPMI 1640 medium supplemented with 5% FBS and 50 μM β-mercaptoethanol. Effector-to-target (E/T) ratios of 200:1, 100:1, 50:1, 25:1, 12.5:1, and 6.25:1 were tested, with each ratio assessed in quadruplicate. After 4 h of incubation, 50 μL of culture supernatant from each well was transferred to a flat-bottom plate. LDH activity in the supernatants was then measured using the CytoTox 96 Non-Radioactive Cytotoxicity Assay (Promega, Madison, WI, USA) according to the manufacturer’s instructions. For phagocytosis assay, apoptotic cells were prepared as previously described (21, 23). Briefly, single-cell suspensions of thymocytes were exposed to UV-C irradiation in PBS for 20 min, washed three times with PBS, and then incubated in serum-free RPMI 1640 medium for 2 h. Peritoneal macrophages were harvested by peritoneal lavage using 3 mL of cold PBS and a 26-G needle. For each well of a 96-well plate, 100 μL of the peritoneal cell suspension was seeded and incubated at 37 °C for 1–2 h to allow macrophage adherence. Non-adherent cells were removed by washing with PBS, and the number of adherent macrophages was determined using a Celigo cytometer (Cyntellect, San Diego, CA, USA).

Apoptotic thymocytes were then co-incubated with macrophages at a ratio of 5:1 in 100 μL of PBS for 1 h at 4 °C. Non-adherent cells were removed by washing with PBS. The remaining cells were fixed with 4% paraformaldehyde, permeabilized with methanol, and stained with hematoxylin and eosin (H&E). Phagocytosis was evaluated by counting 1,000 macrophages per condition. The phagocytic index was expressed as the percentage of macrophages containing at least one apoptotic cell per 100 macrophages counted. For the migration assay, peritoneal macrophages were isolated as described above. Transwell inserts with 5-μm pore-size membranes (Millipore, Bedford, MA, USA) were placed in 24-well plates. Macrophages (5 × 10^4 cells/well) were seeded in the upper chambers in 250 μL of serum-free RPMI 1640 medium, while 750 μL of DMEM supplemented with 10% cosmic calf serum (HyClone, Logan, UT, USA) was added to the lower chambers as a chemoattractant. After incubation at 37 °C for 24 h, non-migrated macrophages remaining on the upper surface of the membranes were removed using cotton swabs. The membranes were then fixed, permeabilized, and stained with hematoxylin. Migrated macrophages were quantified by counting cells in six randomly selected fields per membrane under ×200 magnification.

### 2.7 Reverse-transcription quantitative PCR

Total RNA was extracted using TRIzol reagent, and cDNA was synthesized using a reverse transcription kit (Thermo Fisher Scientific). The mRNA expression levels of IFN-γ, IL-17A, IL-22, IL-23, selected chemokines, and β-actin were quantified by RT-qPCR using the ABI StepOnePlus Real-Time PCR System (Applied Biosystems). The sequences of PCR primers and the corresponding probe reference numbers from the Universal Probe Library (Roche Applied Science, Germany) are listed in **Table 2**. Cytokine expression levels were normalized to β-actin mRNA expression to account for differences in RNA input. Relative mRNA expression was calculated using the 2^−ΔΔCt method.

**Table 2:** The sequences of primers used for qRT-PCR in this study.

Primer name	Sequence (5’-3’)
mouse LGALS1, F	GTAACACCAAGGAAGATGGGACC
mouse LGALS1, R	TCA TGTCCGTCTGGCAGCTTGA
mouse CCL2, F	GCTACAAGAGGATCACCAGCAG
mouse CCL2, R	GTCTGGACCCATTCCTTCTTGG
mouse CCL3, F	GTACCATGACACTCTGCAACC
mouse CCL3, R	GTCAGGAAAATGACACCTGGCTG
mouse CCL5, F	CCTGCTGCTTTGCCTACCTCTC
mouse CCL5, R	ACACACTTGGCGGTTCCTTCGA
mouse CCL17, F	GACCTTCACCTCAGCTTTTG
mouse CCL17, R	CGATGGCATCCCTGGAACAC
mouse CCL22, F	ACTCCTGGTGGCTCTCGTCC
mouse CCL22, R	GACGGTTATCAAACAACGC
mouse CCL20, F	GTACTGCTGGCTCACCTCTG
mouse CCL20, R	CTTCATCGGCCATCTGTCTTGTG
mouse CXCL3, F	CCATCCAGAGCTTGACGGTGAC
mouse CXCL3, R	GGCTCAGCTGGACTTGCCGCTC
mouse CXCL10, F	GCCAACCTTCCGGAAGCCTC
mouse CXCL10, R	CACGTGGGCAGGATAGGCTC
mouse IL-17, F	GCTCCAGAAGGCCC TCAG
mouse IL-17, R	CTTTCCCTCCGCATTGACA
mouse IL-22, F	CGCTGCCCGTCAACACCCGG
mouse IL-22, R	CTGATCTTTAGCACTGACTCCTCG
mouse Loricrin, F	CACATCAGCATCACCTCCTTCC
mouse Loricrin, R	CCTCCTCCACCAGAGGTCTTTC
mouse MIG (CXCL9), F	CCTCTTGGGCATCATCTTCCTG
mouse MIG (CXCL9), R	GGGCTTGGGGCAAACTGTTT
mouse Ngp, F	GACTGCGACTTCCTGGAGGATG
mouse Ngp, R	GTATCCTCTCGACTGCAATCCCT
mouse S100A7, F	GAGGAGTTGAAAGCTCTGCTCTTG
mouse S100A7, R	GTGATGTAGTATGGCTGCCTGCG
mouse S100A8, F	CACCATGCCCCCTCTACAAGAATGA
mouse S100A8, R	CTCTGCTACTCCTTGTGGCTG

F, forward; R, reverse.

### 2.8 Statistical analysis

Data are presented as the mean ± SEM. Differences in PASI-like scores were analyzed using repeated-measures analysis of variance (ANOVA). All other data were analyzed by two-way ANOVA followed by Bonferroni’s *post hoc* test. A p value of < 0.05 was considered statistically significant. All statistical analyses were performed using GraphPad Prism version 6.0 (GraphPad Software, San Diego, CA, USA).

## 3 Results

### 3.1 Gal-1 expression is increased in human psoriatic lesions and in IMQ-treated murine skin

Gal-1 immunoreactivity was detected in dermal and stromal compartments of control skin and was more extensive in lesional skin from patients with psoriasis (**Figures 1a, b**). In the mouse model, IMQ treatment increased the number of Gal-1-positive cells in dorsal skin and increased Gal-1 transcript abundance relative to untreated B6 controls (**Figures 1c–e**). These findings establish that Gal-1 expression is enhanced in human psoriasis lesions and in IMQ-induced psoriasiform skin inflammation.

**Figure 1:**
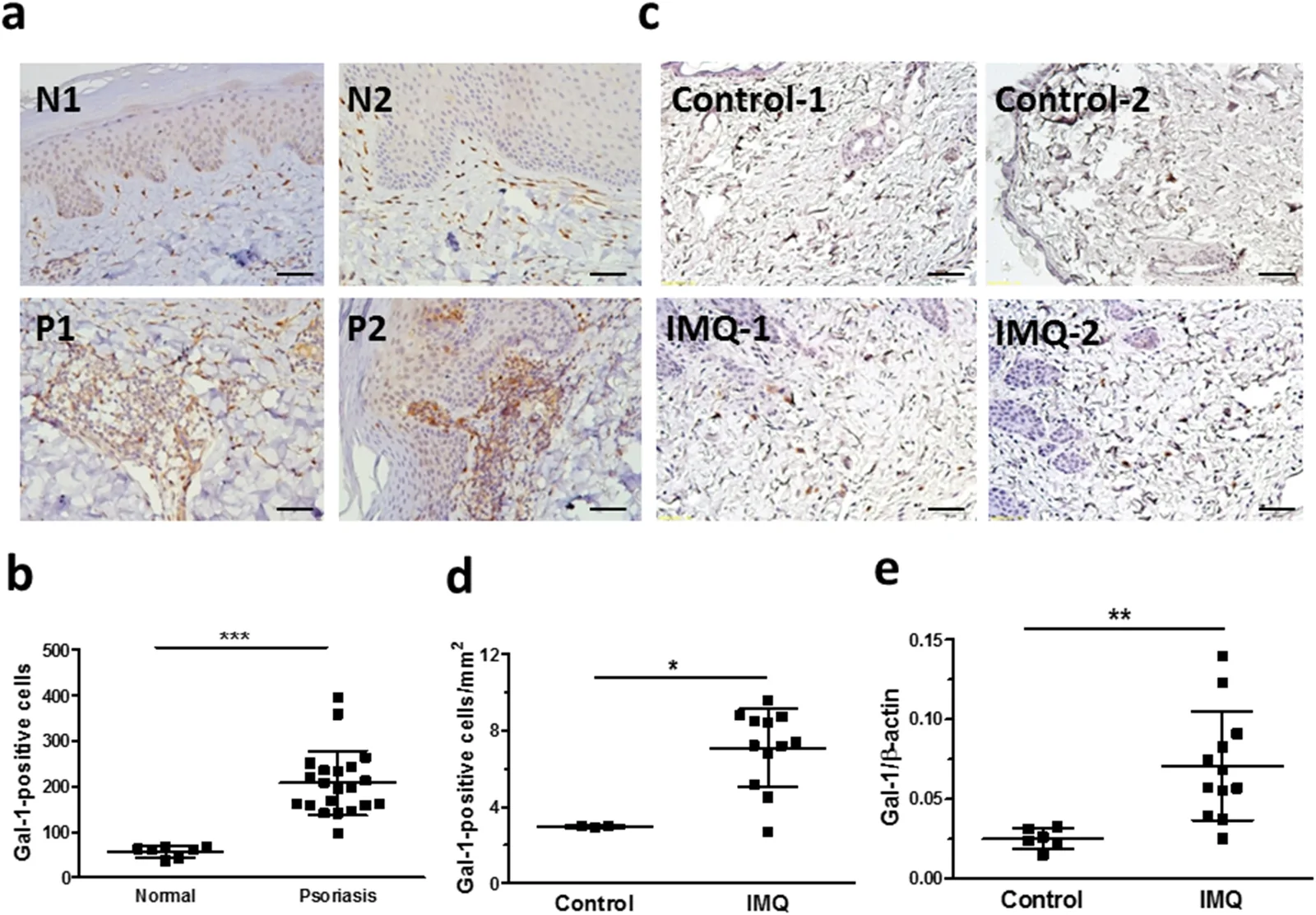
Gal-1 expression is increased in human psoriatic lesions and in imiquimod (IMQ)-treated murine skin. **(a)** Representative Gal-1 immunohistochemistry in normal skin (N1 and N2) and lesional skin from patients with psoriasis (P1 and P2). **(b)** Quantification of Gal-1-positive cells in normal and psoriatic skin; the supplied figure indicates n = 7 normal and n = 21 psoriasis specimens. **(c)** Representative Gal-1 immunostaining in dorsal skin from untreated B6 mice (control) and B6 mice treated with imiquimod (IMQ). **(d)** Quantification of Gal-1-positive cells in murine skin (control, n=6; IMQ, n=12). **(e)** Gal-1 mRNA expression in murine skin measured by RT-qPCR and normalized to β-actin. Data are mean ± SEM. Scale bars indicate 100 µm. *p < 0.05; **p < 0.01; ***p < 0.001.

### 3.2 Gal-1 deficiency attenuates IMQ-induced psoriasiform skin inflammation

Topical IMQ induced erythema, scaling, and skin thickening in both genotypes; however, all clinical PASI-like scores and the cumulative score were lower in Gal-1-deficient mice than in IMQ-treated B6 mice (**Figures 2a, b**). Histological examination on day 6 showed less epidermal hyperplasia, acanthosis, and parakeratosis in Gal-1-deficient mice (**Figure 2c**). Quantification confirmed reductions in both layer-based and micrometer-based measures of acanthosis and parakeratosis in IMQ-treated Gal-1-deficient mice (**Figure 2d**). These results indicate that endogenous Gal-1 contributes to the development of IMQ-induced psoriasiform skin inflammation.

**Figure 2:**
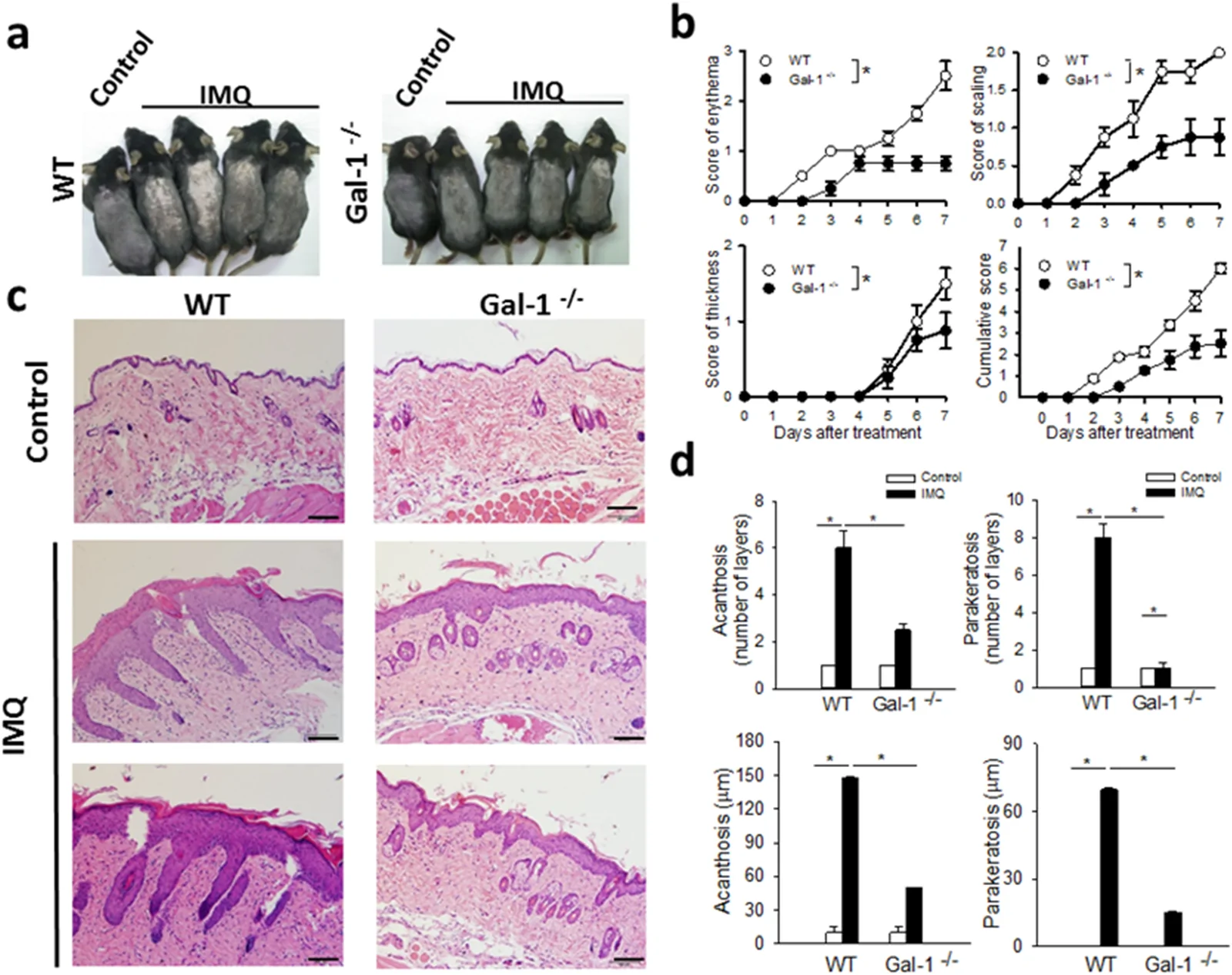
Gal-1 deficiency attenuates IMQ-induced psoriasiform skin inflammation. **(a)** Representative macroscopic images of dorsal skin from wild-type (WT) and Gal-1^−/−^ mice with or without IMQ treatment for six consecutive days. **(b)** Daily erythema, scaling, thickening, and cumulative Psoriasis Area and Severity Index (PASI)-like scores. **(c)** Representative H&E-stained dorsal-skin sections collected on day 6; images show two IMQ-treated samples per genotype. **(d)** Quantification of acanthosis and parakeratosis, expressed as keratinocyte-layer number and epidermal thickness (µm), as labeled. Data are mean ± SEM (n=4). Scale bars indicate 200 µm. *p < 0.05.

### 3.3 Gal-1 deficiency is associated with reduced systemic innate and adaptive immune activation after IMQ treatment

IMQ treatment increased spleen and draining lymph-node size and weight in B6 mice, whereas these systemic changes were attenuated in Gal-1-deficient mice (**Figures 3a, b**). Flow-cytometric analysis showed that IMQ-treated B6 mice had increased proportions of macrophages, NK cells, and pDCs in the spleen and/or draining lymph nodes; these increases were diminished in IMQ-treated Gal-1-deficient mice (**Figure 3c**). IMQ treatment was also associated with changes in IFN-γ-, IL-4-, IL-17-, and Foxp3-positive CD4+ T-cell populations in the spleen and draining lymph nodes, with significantly lower IFN-γ- and IL-17-associated responses in Gal-1-deficient mice in the comparisons indicated in **Figure 3d**. These data support an association between Gal-1 deficiency and attenuated systemic innate and T-cell-associated immune activation after IMQ treatment.

**Figure 3:**
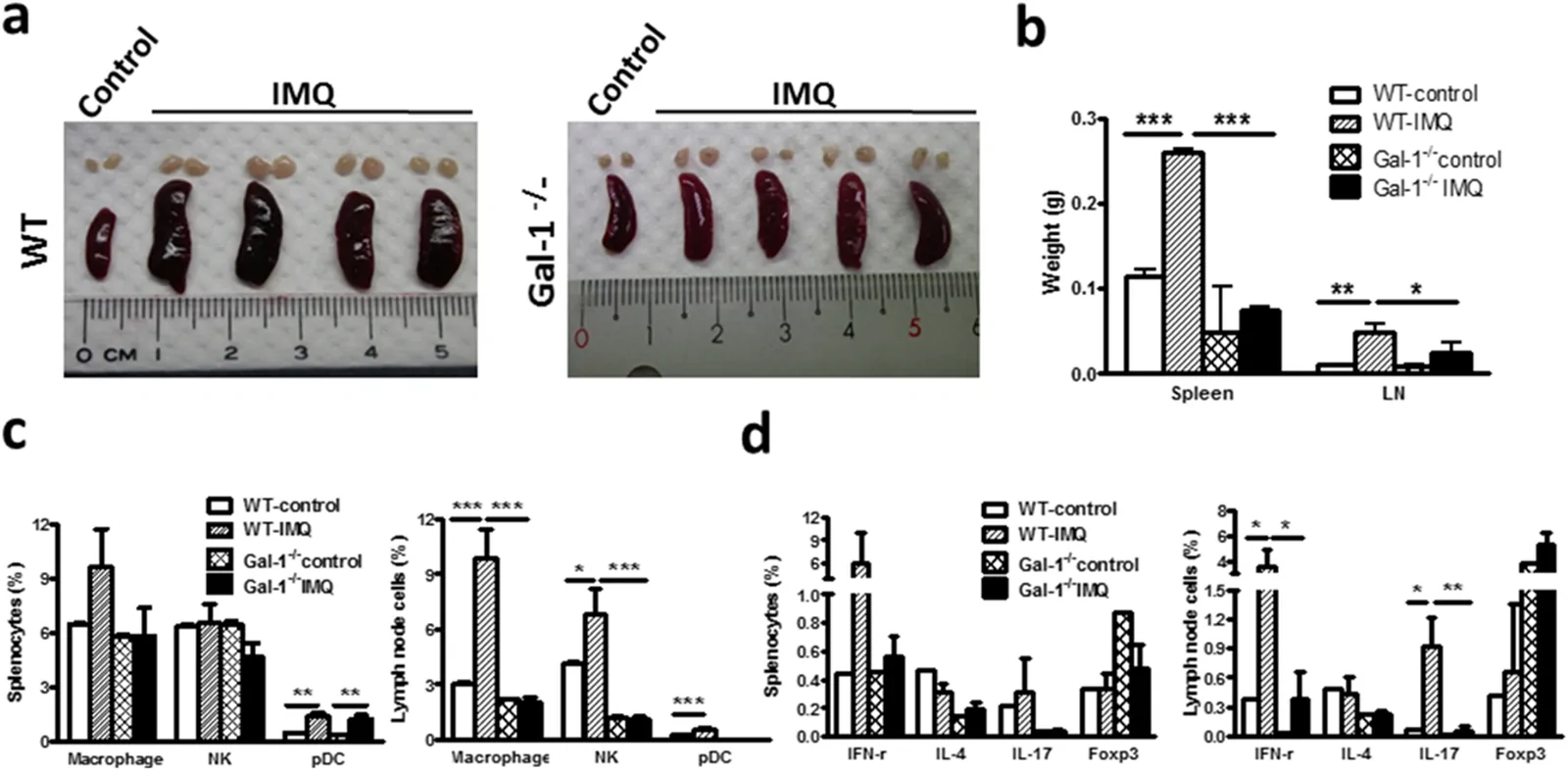
Gal-1 deficiency is associated with reduced systemic immune activation after IMQ treatment. **(a)** Representative spleens and draining lymph nodes from untreated and IMQ-treated WT and Gal-1^−/−^ mice on day 6. **(b)** Spleen and lymph-node weights. **(c)** Flow-cytometric quantification of macrophages, NK cells, and pDCs in the spleen and draining lymph nodes. **(d)** Flow-cytometric analysis of IFN-γ-, IL-4-, IL-17-, and Foxp3-positive CD4+ T-cell populations in the spleen and draining lymph nodes. Data are mean ± SEM (n = 4). *p < 0.05; **p < 0.01; ***p < 0.001.

### 3.4 Gal-1 deficiency selectively reduces IMQ-associated CXCL10 induction in skin

We next quantified selected inflammatory and epidermal-response transcripts in dorsal skin. IMQ treatment increased CCL5, CCL17, CXCL10, IL-22, and S100A8 expression in B6 mice (**Figure 4**). Compared with IMQ-treated B6 mice, IMQ-treated Gal-1-deficient mice showed significantly lower induction of CCL5, CXCL10, and S100A8; CCL17 was also lower, whereas the difference in IL-22 was not statistically significant in the supplied figure. These data indicate that Gal-1 deficiency is associated with reduced expression of selected chemokines and inflammatory/epidermal-response genes in IMQ-inflamed skin.

**Figure 4:**
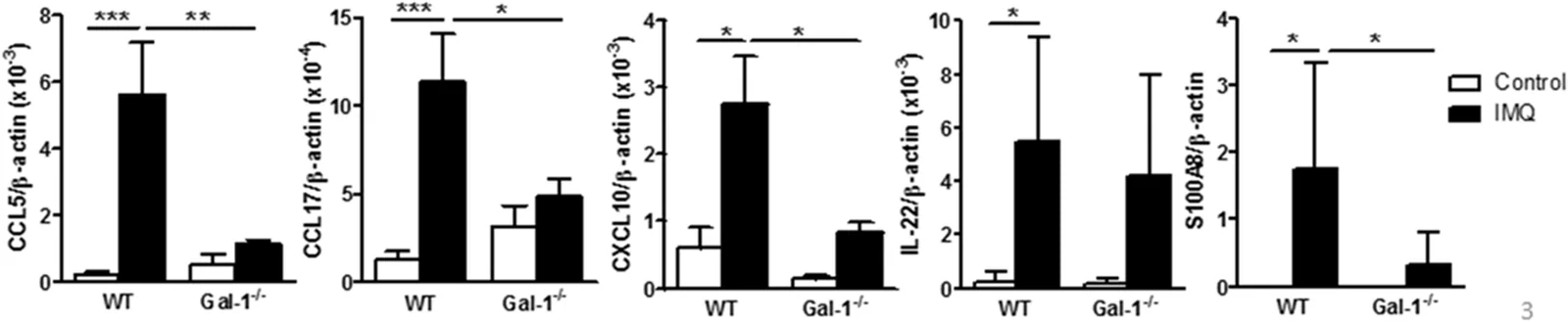
Gal-1 deficiency reduces selected IMQ-induced inflammatory transcripts in skin. Dorsal skin was collected on day 6 from untreated and IMQ-treated WT and Gal-1^−/−^ mice. CCL5, CCL17, CXCL10, IL-22, and S100A8 mRNA expression was measured by RT-qPCR, normalized to β-actin, and expressed as shown on the y-axes. Data are mean ± SEM (n=4-8). *p < 0.05; **p < 0.01; ***p < 0.001.

### 3.5 Gal-1 deficiency reduces macrophage accumulation in inflamed skin and impairs macrophage migration

Immunohistochemical analysis demonstrated marked accumulation of CD49b-positive cells and Mac-3-positive macrophages in IMQ-treated B6 skin compared with untreated B6 skin. Both readouts were significantly lower in IMQ-treated Gal-1-deficient skin (**Figures 5a–d**). Functional analyses showed no statistically significant genotype-related difference in NK-cell cytotoxicity or in the macrophage phagocytosis readout (**Figures 6a–c**). In contrast, the skin-explant migration assay demonstrated substantially fewer migrating macrophages in settings involving Gal-1-deficient macrophages and/or Gal-1-deficient skin (**Figure 6d**). Together, these observations are consistent with a role for Gal-1 in immune-cell accumulation in inflamed skin and macrophage migratory responses, rather than cytotoxic or phagocytic capacity.

**Figure 5:**
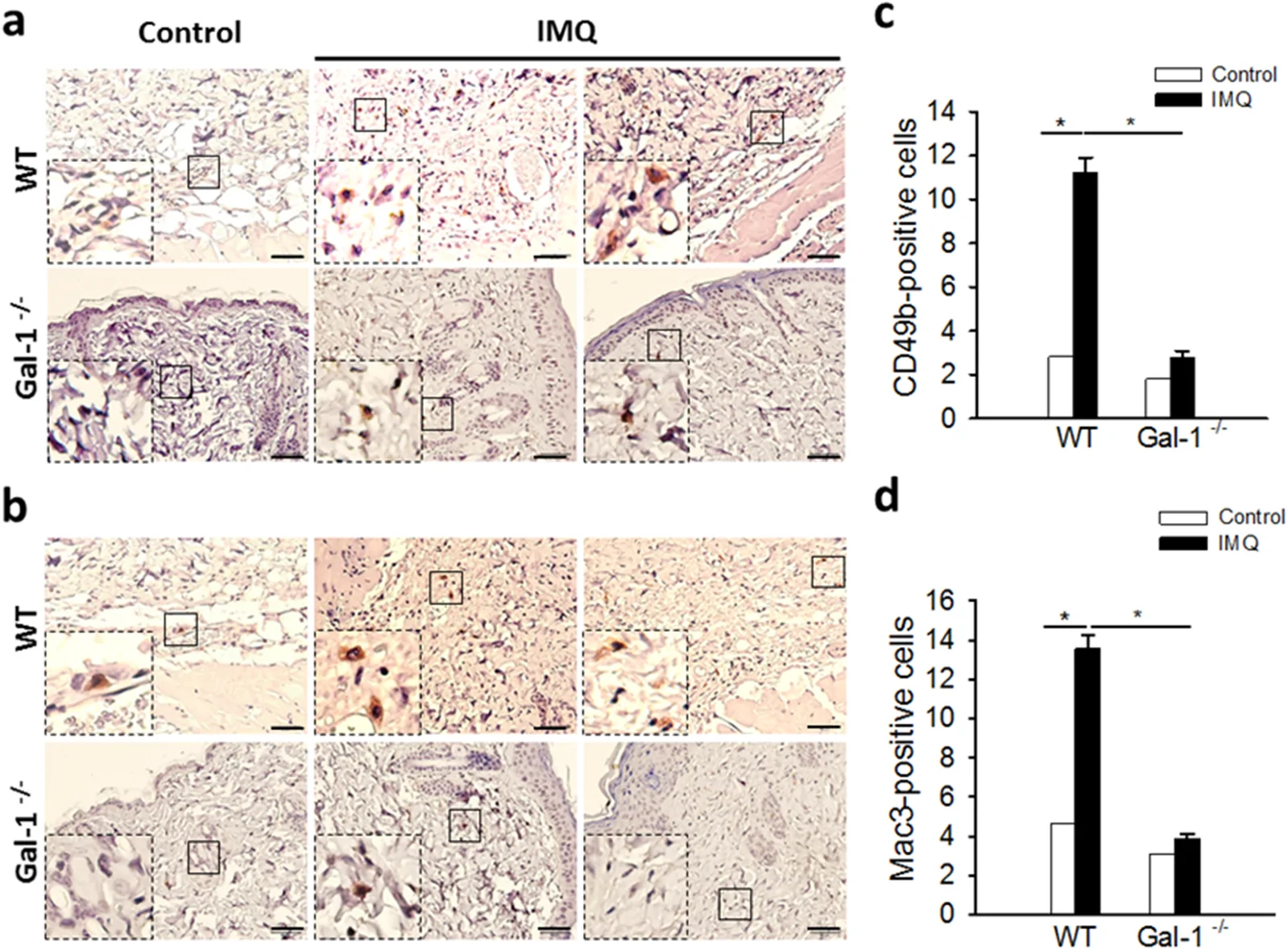
Gal-1 deficiency reduces CD49b-positive and Mac-3-positive cell accumulation in IMQ-treated skin. **(a)** Representative CD49b immunohistochemistry in dorsal skin from untreated and IMQ-treated WT and Gal-1−/− mice. **(b)** Representative Mac-3 immunohistochemistry in the same experimental groups. Insets show higher-magnification regions indicated by boxes. **(c, d)** Quantification of CD49b-positive and Mac-3-positive cells, respectively. Data are mean ± SEM (n=4). Scale bars indicate 20 µm. *p < 0.05.

**Figure 6:**
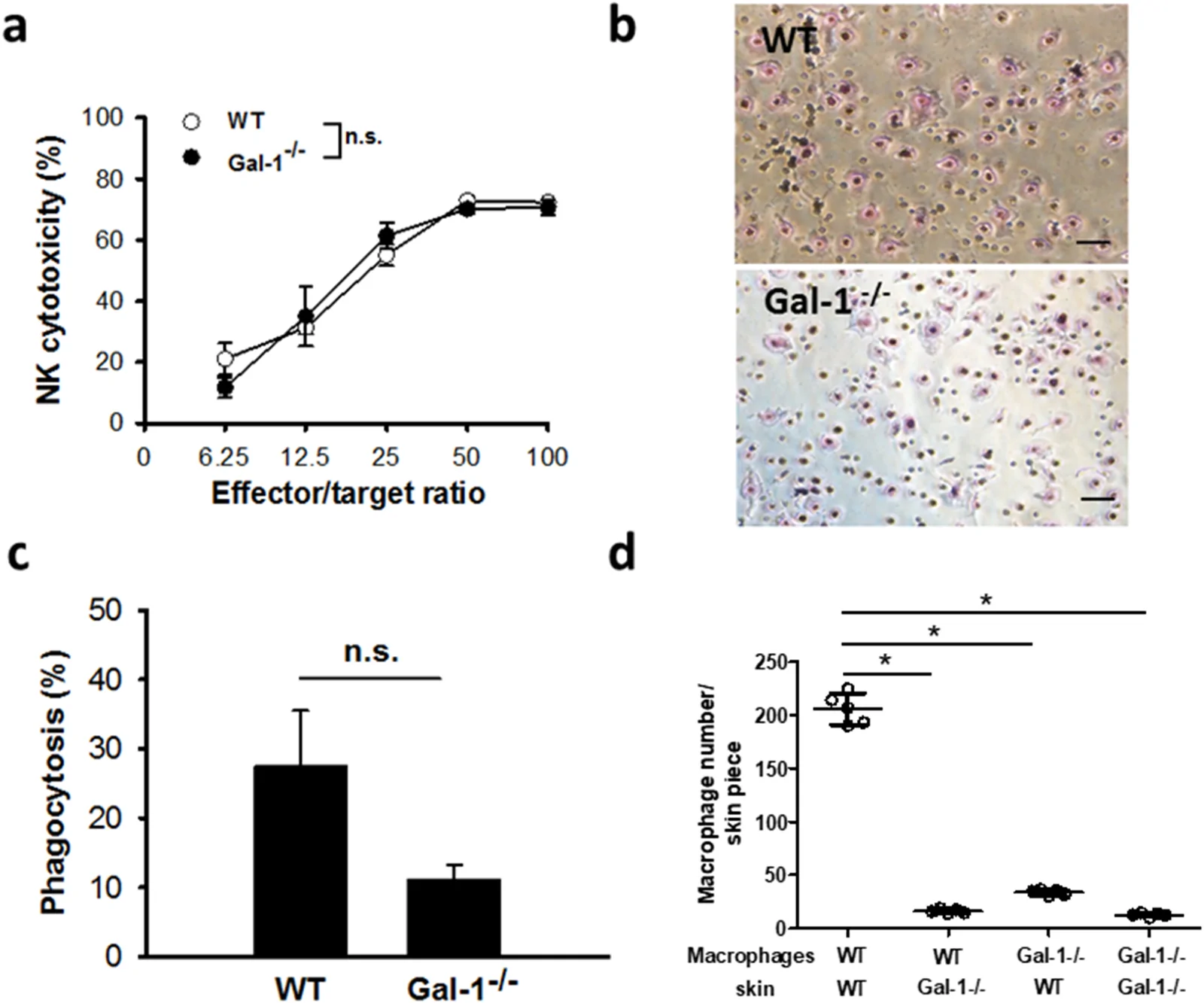
Gal-1 deficiency selectively impairs macrophage migration without demonstrable changes in NK-cell cytotoxicity or macrophage phagocytosis. **(a)** NK-cell cytotoxicity of splenocytes from WT and Gal-1−/− mice against YAC-1 target cells at the indicated effector-to-target ratios. **(b)** Representative images from the macrophage phagocytosis assay. **(c)** Quantification of macrophage phagocytosis. **(d)** Macrophage migration toward skin explants in the four macrophage/skin genotype combinations labeled beneath the x-axis. Data are mean ± SEM (n=5). Scale bars indicate 20 µm. n.s., not significant; *p < 0.05.

### 3.6 Recombinant Gal-1 enhances macrophage migration

To determine whether exogenous Gal-1 could modulate macrophage migration, peritoneal macrophages from B6 and Gal-1-deficient mice were pretreated with recombinant Gal-1 and assessed in transwell assays. Recombinant Gal-1 increased the number of migrated macrophages in a dose-responsive manner, with a statistically significant effect at 0.5 μg/mL in the comparisons shown (**Figures 7a, b**). When migration was normalized to the untreated genotype-matched control, recombinant Gal-1 increased the relative migration index in both genotypes (**Figure 7c**). These findings support a promigratory effect of recombinant Gal-1 under the conditions tested.

**Figure 7:**
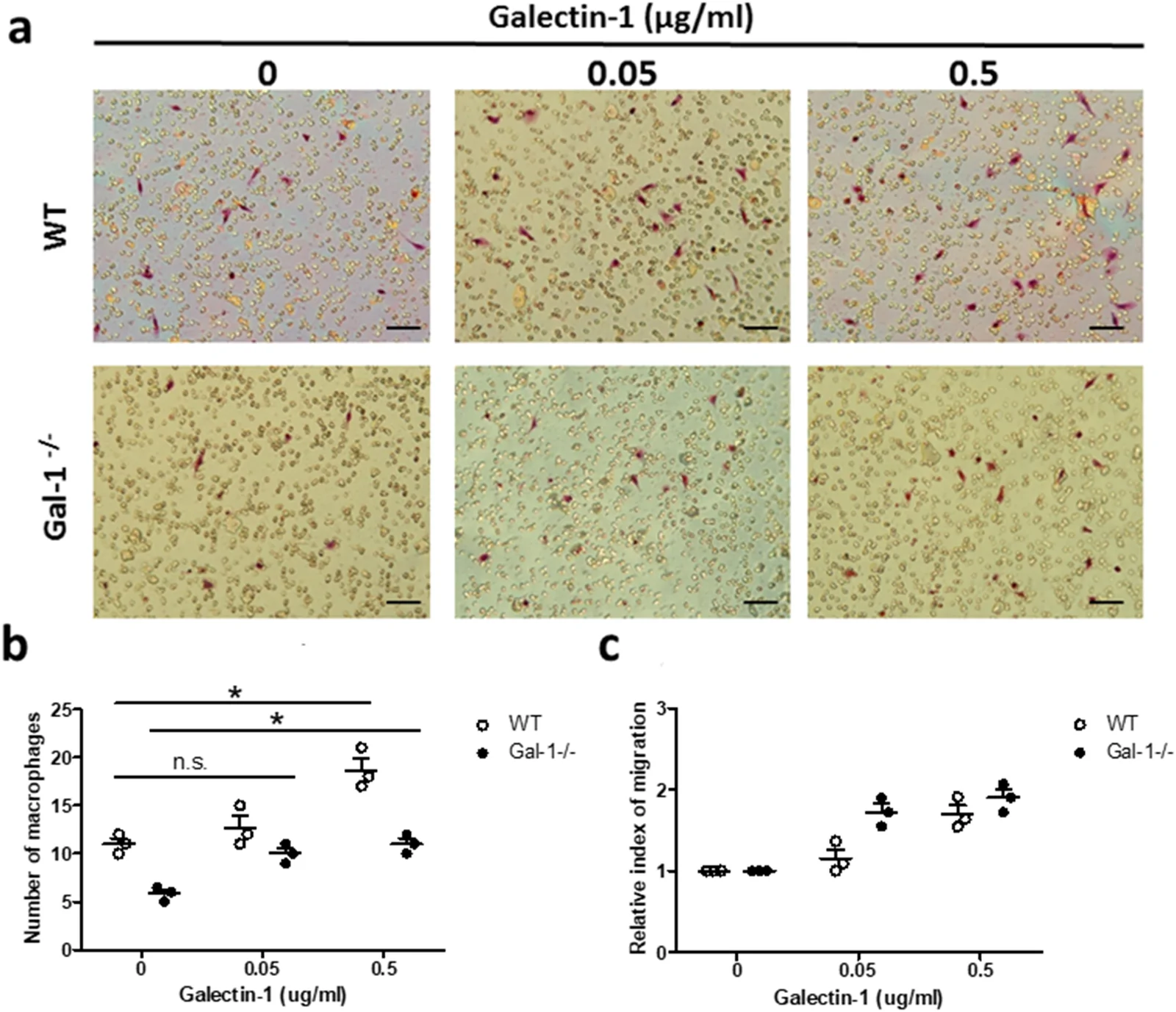
Recombinant Gal-1 enhances macrophage migration in transwell assays. **(a)** Representative images of migrated peritoneal macrophages from WT and Gal-1^−/−^ mice after pretreatment with recombinant Gal-1 (0, 0.05, or 0.5 μg/mL). **(b)** Quantification of migrated macrophages. **(c)** Relative migration normalized to the untreated genotype-matched control. Data are mean ± SEM (n = 3). n.s., not significant; *p < 0.05.

## 4 Discussion

Gal-1 is often described as an immunoregulatory lectin with anti-inflammatory effects, particularly through apoptosis of activated T cells and induction of tolerogenic immune programs (8–10, 31–33). However, the present findings emphasize that Gal-1 biology is highly context dependent. In the IMQ model, Gal-1 deficiency reduced clinical and histological disease severity, lymphoid-organ enlargement, cutaneous macrophage accumulation, and selected inflammatory transcripts. These observations do not negate the immunosuppressive activity of Gal-1 in other autoimmune or inflammatory settings; rather, they indicate that Gal-1 can function as either a brake or an amplifier of inflammation depending on the dominant cell population, tissue microenvironment, and stage of the inflammatory response.

The increased Gal-1 immunoreactivity in human psoriatic lesions is consistent with the concept that galectins participate in the inflammatory and remodeling responses of diseased skin. Earlier studies of psoriatic skin reported altered expression of several galectin family members and changes in galectin-binding glycoconjugates, supporting the presence of a disease-associated glycan-lectin microenvironment (34, 35). More recent analyses have also suggested that Gal-1 and Gal-3 expression patterns differ between inflammatory skin diseases and may be influenced by the local balance of keratinocyte activation, dermal inflammation, and immune-cell infiltration (36, 37). Therefore, the increase in Gal-1 observed in the present study should be interpreted not simply as a marker of inflammation, but as part of a broader tissue response in which epithelial, stromal, endothelial, and immune compartments may all contribute to disease amplification or resolution.

A major implication of our study is that Gal-1 may regulate psoriasiform inflammation through effects on inflammatory-cell recruitment. Psoriasis is sustained by reciprocal interactions between keratinocytes and immune cells, in which cytokines and chemokines produced by activated keratinocytes, dendritic cells, T cells, and myeloid cells reinforce leukocyte influx and epidermal hyperplasia (4–6, 38, 39). In this context, reduced accumulation of Mac-3-positive macrophages in IMQ-treated Gal-1-deficient skin provides a plausible cellular explanation for the attenuated phenotype. Macrophages are not passive bystanders in psoriasiform inflammation; they can produce or amplify TNF, IL-1-family cytokines, IL-23-related responses, and chemokine networks that sustain Th17- and keratinocyte-driven inflammatory loops (24–27, 40). Thus, reduced macrophage recruitment may indirectly contribute to the lower epidermal thickening and inflammatory transcript expression observed in Gal-1-deficient mice.

The macrophage migration assays further support a promigratory role of Gal-1. Gal-1 has been shown to stimulate monocyte chemotaxis through p44/42 MAP kinase- and pertussis-toxin-sensitive pathways and to regulate extracellular-matrix-dependent migration of dendritic cells and other cell types (16, 17). Our finding that recombinant Gal-1 enhanced macrophage migration in cells from both wild-type and Gal-1-deficient mice is consistent with a direct extracellular effect of Gal-1 on migratory behavior. Nevertheless, the *in vivo* mechanism remains unresolved. Gal-1 may act on macrophages directly by modulating glycosylated surface receptors, integrin-dependent adhesion, or chemokine-receptor signaling; alternatively, Gal-1 may alter the inflamed skin microenvironment and thereby secondarily change macrophage recruitment. Distinguishing these possibilities will require macrophage-specific Gal-1 manipulation, adoptive-transfer approaches, or bone-marrow chimeric models.

The reduction of CXCL10 expression in Gal-1-deficient skin is also mechanistically relevant but should be interpreted cautiously. CXCL10 is a CXCR3 ligand involved in effector T-cell generation and trafficking and has been linked to active psoriatic lesions and treatment-responsive inflammatory activity (28–30, 41). In psoriatic skin, IFN-γ and TNF-related signals can induce keratinocytes and myeloid cells to produce CXCL10 and other chemokines that attract CXCR3-expressing T cells, NK cells, and monocyte-lineage cells (28–30, 38, 39, 41). The present data therefore support an association between Gal-1 deficiency and reduced CXCL10-associated chemotactic signaling. However, because the current experiments do not include CXCL10 blockade, CXCR3 inhibition, or Gal-1 rescue with downstream chemokine analysis, CXCL10 should be discussed as an associated molecular change rather than as a proven mediator of the Gal-1 effect.

The qPCR data also showed reduced CCL5 and S100A8 expression in IMQ-treated Gal-1-deficient skin. This pattern is potentially important because CCL5 participates in leukocyte recruitment, whereas S100A8 is part of the S100A8/A9 inflammatory alarmin complex that is strongly linked to epidermal activation and neutrophil/myeloid inflammation in psoriatic lesions (38, 39, 42). IL-22, by contrast, did not show a clear genotype-dependent reduction in the supplied dataset, even though IL-22 is a well-established contributor to epidermal responses in psoriasis (3). These results suggest that Gal-1 deficiency does not globally suppress all psoriasiform inflammatory pathways. Instead, Gal-1 appears to influence a subset of chemokine and myeloid-associated responses that may converge on inflammatory-cell recruitment and tissue inflammation.

The systemic immune findings should also be interpreted in light of the acute and innate-biased nature of the IMQ model. IMQ-induced inflammation involves TLR7/8-related innate activation, pDC activation, IL-23/IL-17-axis engagement, and secondary adaptive immune changes (21, 22). The lower spleen and lymph-node enlargement and altered innate and T-cell-associated populations in Gal-1-deficient mice indicate that Gal-1 deficiency modifies both local and systemic responses to topical IMQ. Importantly, we cannot distinguish whether these systemic changes result from a direct effect of Gal-1 deficiency on immune cells or occur secondarily to the reduced severity of cutaneous inflammation. However, systemic effects in this model do not necessarily mean that Gal-1 has the same role in chronic human plaque psoriasis, where genetic susceptibility, tissue-resident immune memory, vascular remodeling, neuroimmune factors, metabolic comorbidities, and repeated environmental triggers contribute to disease persistence (31, 32, 43).

From a translational perspective, the data support Gal-1 as a potential therapeutic target for modulating inflammatory-cell recruitment in psoriasis-like skin inflammation. This possibility is attractive because current biologics efficiently target IL-23 and IL-17, yet upstream tissue-retention and leukocyte-recruitment mechanisms remain less completely defined (5, 6, 32, 33). However, Gal-1-targeted intervention would require careful design. Because Gal-1 can promote tolerogenic circuits and T-cell apoptosis in other settings (8–10, 31–33), systemic Gal-1 blockade could have undesirable consequences. Local delivery, cell-type-selective targeting, or pathway-specific modulation may therefore be more realistic than broad systemic inhibition. In addition, whether Gal-1 is pathogenic, compensatory, or both may vary according to disease stage and cellular source.

The study has limitations. First, the IMQ model is a useful and widely adopted model for studying psoriasiform inflammation because it reproduces key pathological and immunological features of psoriasis, including epidermal hyperplasia, inflammatory-cell infiltration, and IL-23/IL-17-axis activation (22, 44). Nevertheless, this acute inducible model does not fully capture the chronic relapsing nature, genetic predisposition, environmental contributions, treatment response, and heterogeneous immune-cell networks that characterize human psoriasis (44, 45). Second, the use of global Gal-1-deficient mice does not identify the pathogenic cellular source of Gal-1. Third, the current figures show an association among Gal-1 deficiency, CXCL10 expression, and macrophage accumulation, but do not prove a causal Gal-1-CXCL10-macrophage axis. Conditional knockout, bone-marrow chimera, local Gal-1 rescue, recombinant Gal-1 neutralization, macrophage-transfer, and CXCR3-pathway experiments would be needed to define this mechanism. Fourth, although the skin-explant migration assay provides a tissue-associated context, it does not fully recapitulate the cytokine- and keratinocyte-rich inflammatory microenvironment of psoriasiform skin. Future studies incorporating activated keratinocytes, reconstructed inflammatory skin systems, or other tissue-relevant models will be required to further define how Gal-1 influences macrophage recruitment under inflammatory conditions.

In conclusion, Gal-1 deficiency attenuated IMQ-induced psoriasiform inflammation and was associated with reduced macrophage recruitment and migration, attenuated systemic immune responses, and reduced expression of selected chemokine and myeloid-associated inflammatory genes. These findings expand the context-dependent functions of Gal-1 and support further investigation of its role in regulating inflammatory-cell recruitment in psoriasis-like skin disease.

## Data Availability

The original contributions presented in the study are included in the article/supplementary material. Further inquiries can be directed to the corresponding author.
